# Atomic-level flatness on oxygen-free copper surface in lapping and chemical mechanical polishing

**DOI:** 10.1039/d2na00405d

**Published:** 2022-08-22

**Authors:** Dongdong Liu, Zhenyu Zhang, Jiajian Feng, Zhibin Yu, Fanning Meng, Guanghong Xu, Jianmei Wang, Wei Wen, Wei Liu

**Affiliations:** Key Laboratory for Precision and Non-Traditional Machining Technology of Ministry of Education, Dalian University of Technology Dalian 116024 China zzy@dlut.edu.cn; Foshan Tanzhituo Advanced Intelligent Equipment Co., Ltd Foshan 528203 China; Dianxi Research Institute of Dalian University of Technology Baoshan 678000 China; Division of Energy Research Resources, Dalian National Laboratory for Clean Energy, Dalian Institute of Chemical Physics, Chinese Academy of Sciences Dalian Liaoning 116023 China; Engineering Research Center Heavy Machinery Ministry of Education, Taiyuan University of Science and Technology Taiyuan 030024 China; College of Mechanical and Electrical Engineering, Hainan University Haikou 570228 China

## Abstract

Oxygen-free copper (OFC) serves as a core component of high-end manufacturing, and requires high surface quality. It is always a significant challenge to manufacture high-quality atomic-level surfaces. In this study, SiO_2_ nanospheres with good dispersibility were prepared and a late-model environmentally friendly chemical mechanical polishing (CMP) slurry was developed. The CMP slurry consists of SiO_2_ nanospheres, CeO_2_ nanospheres, H_2_O_2_, NaHCO_3_, polyaspartic acid and deionized water. After CMP, the average roughness (Sa) of the OFC wafer reached 0.092 nm with an area of 50 × 50 μm^2^. Atomic-level flatness on the oxygen-free copper surface was acquired, which has never been reported before. Moreover, the mechanical removal mechanism of abrasive particles and the chemical reactions during lapping and CMP are proposed in detail. The thickness and composition of the damaged layer after lapping and polishing were analyzed. The lapping-damaged layer consists of a lattice distortion region, moiré fringes, grain boundary, superlattice and edge dislocations, and the polishing-damaged layer contains a handful of stacking faults with single-layer or multi-layer atoms. The chemical action involves three reactions: oxidation, corrosion and chelation. The processing method and its mechanistic explanation pave the way for the fabrication of high-performance OFC surfaces for use in vacuum, aerospace, military and electronic industries.

## Abstract 2

Schematic diagram of chemical mechanical polishing on oxygen-free copper: (a) the pristine surface prior to CMP, (b) the chemical reaction, (c) mechanical removal and (d) atomic-level ultra-smooth surface after CMP.

## Graphical abstract

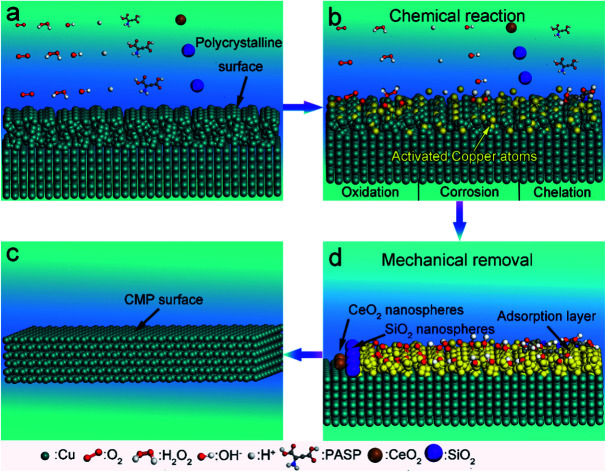

## Introduction

Oxygen-free copper has the advantages of good thermal conductivity, good electrical conductivity, low resistivity, corrosion resistance, non-magnetic nature, easy processing, low cost and low hydrogen permeability,^1–4^ and is widely used in the fabrication of precision electrodes, optical materials, catalytic substrates, and military and vacuum electronic devices and components.^5–7^ The special working conditions of OFC components require it to have low damage, high dimensional accuracy and high surface flatness. At present, the polishing methods used to obtain low damage and high surface accuracy include electrochemical polishing,^8^ plasma-assisted chemical polishing,^9^ bowl-feed polishing,^10^ and chemical mechanical polishing.^11^ All of the above methods can reduce the surface roughness and improve the machined surface quality, but some of them still have inevitable disadvantages. In electrochemical polishing, the electrolytes are generally corrosive and toxic, and the application range of electrochemical polishing is limited.^12^ CMP technology is a critical process for global planarization.^13,14^

The surface roughness and polishing rate are two major evaluation indexes of CMP performance. In order to ensure surface quality while increasing the polishing rate, many factors affecting surface quality have been studied for decades; among them, the CMP slurry is the most influencing parameter. Xu *et al.*^15^ investigated the effect of slurry components on the CMP performance of copper. By suitable configuration of the slurry components and optimal choice of the concentrations of the constituents, a surface roughness of less than 2.5 nm was acquired. Zhang *et al.*^16^ developed the novel environmentally friendly chemical mechanical polishing of copper. After chemical mechanical polishing, the surface roughness (Ra) was 0.444 nm. Liu *et al.*^17^ explored the thermal effects in the CMP process by considering the slurry, pad, and wafer. A temperature of approximately 40 °C was demonstrated to be a suitable choice. The surface roughness (Ra) value of the polished copper wafers was 0.5 nm. Gao *et al.*^18^ synthesized a series of fine-structured rod-shaped silica (RmSiO_2_)-based abrasives with controllable sizes and diverse ordered mesoporous structures *via* a soft template approach, and successfully applied these in a sustainable polishing slurry to improve the surface quality of cadmium zinc telluride (CZT) wafers. Jeong *et al.*^19^ studied a mixed abrasive slurry (MAS), which is a non-traditional slurry with two differently sized abrasives, and controlled the mixing ratio in order to improve the CMP removal rate (RR). None of the above results could attain a surface with atomic flatness. Moreover, the CMP mechanism has also been studied by a great many scholars to acquire flattened and low-damaged surfaces. For example, Choi *et al.*^20^ proposed a novel mechanical material removal mechanism based on the hardness of copper at very small scales to explain that the majority of material removal during copper CMP can be attributed to the removal of copper through abrasion rather than corrosion. Yun *et al.*^21^ designed a novel one-step Cu-film CMP process using a chemical and mechanical dominant CMP mechanism. Moreover, the CMP mechanism involves both chemical-oxidation and etching dominant polishing, which simultaneously occur during Cu CMP. Kawaguchi *et al.*^22^ developed a tight-binding quantum chemical molecular dynamics (TB-QCMD) code to study the chemical reactions during CMP and revealed the chemical reactions of aqueous slurries and the mechanical friction of abrasive grains. To better understand the copper CMP mechanism, it is crucial to investigate the mechanical damage and chemical reactions that occur during CMP.

In this work, to meet the excellent performance of polishing requirements, homogeneous SiO_2_ nanospheres were synthesized. The SiO_2_ nanospheres were used to formulate a novel non-corrosive polishing slurry. After lapping and CMP, the surface morphology and surface roughness were characterized, respectively. The area roughness (Sa) value is less than 0.1 nm. Ultra-smooth and atomic-level surface flatness was achieved. Moreover, the mechanical damage caused by the SiO_2_ nanospheres and the chemical action of the CMP slurry were analyzed. Three types reactions, namely oxidation, corrosion and chelation, were suggested during CMP.

## Materials and methods

OFC specimens with dimensions of 70 × 10 × 5 mm^3^ were provided by Sanle Electronic Information Industry Group Co., Ltd, China, which were cut into 10 × 10 × 5 mm^3^ square wafers by wire cutting. Three OFC wafers were evenly glued to the sample loading plate. An automatic pressure grinding and polishing machine (UNIPOL-1200S, Shenyang Kejing Automation Equipment Co., Ltd, China) was employed to process the wafers. Firstly, self-adhesive SiC sandpaper was fixed on a lapping and polishing plate; sheets of sandpaper with different grit sizes (800, 1000, 2000, 4000) were then used to remove oil stains and the oxide layer and to improve the surface quality of the wafers, with lapping durations of 2, 2, 4 and 6 min, respectively. During lapping, the lapping pressure was set to 12 kPa and the lapping plate rotational speed to 50 rpm by a program. Deionized water acted as the lapping slurry. After lapping, the OFC wafers were cleaned with deionized water and dried with compressed nitrogen.

Next, the as-developed novel slurry for chemical mechanical polishing was prepared; the SiC sandpaper was replaced with a nubuck leather polishing pad. During CMP, the rotational speeds of both the polishing plate and OFC wafers were 60 rpm, the pressure was 15 kPa, the flow rate of the polishing slurry was 8 ml min^−1^ and the polishing time was 20 min. After CMP, deionized water was used to clean the residual polishing slurry on the surface and the surface was dried with nitrogen.

The surfaces processed by lapping and polishing were characterized by scanning electron microscopy (FEI Quanta 650 FEG, Thermo Scientific Company, USA). The surface quality and geometric accuracy were measured by 3D optical surface profilometry (Zygo NewView 9000, USA) and atomic force microscopy (Nanowizard4XP, Bruker, Germany). The damaged forms of the surface after lapping and polishing were analyzed by a Titan Themis G3 environmental transmission electron microscope (ETEM, Thermo Scientific Company, USA). Cross-sectional TEM specimens were prepared by a focused ion-beam (Helios G4 UX, Thermo Scientific Company, USA) from the lapping and polishing surfaces. Firstly, silver conductive paint (SCP) was applied on the surface of the processed wafers to avoid damage caused by the preparation of the TEM samples. Secondly, a Pt protective layer with dimensions of 10 × 2 × 2 μm^3^ was deposited on the coatings. Then, the specimen was first milled on both sides by a 30 kV ion beam current to make sure it was thin enough to be transparent for TEM. Then, a specimen was cut through from the wafer and picked up on the copper grid with the manipulator of the FIB equipment. Lastly, the specimens were milled, thinned and the amorphous substances were removed with 30 kV, 8 kV and 2 kV, respectively. The surfaces elements of the OFC wafers before and after CMP were investigated by XPS (ESCALAB 250Xi, Thermo Scientific Company, USA).

## Results and discussion

**Fig. 1:**
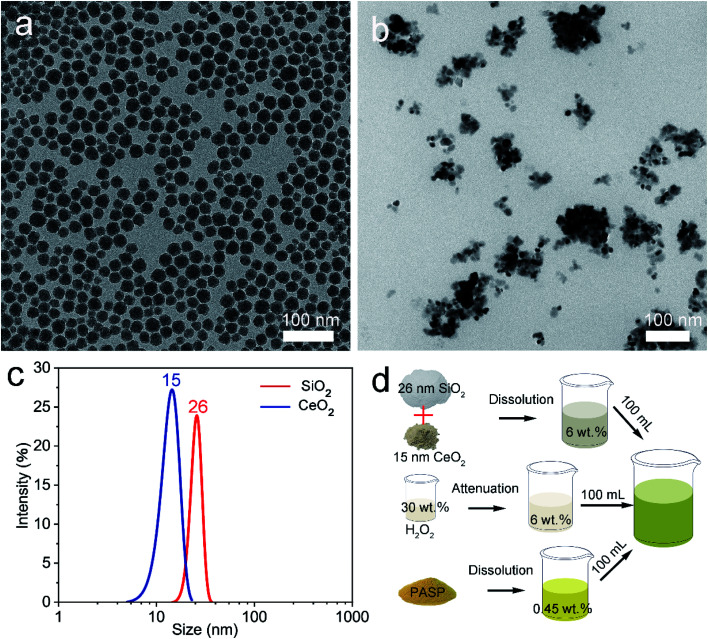
TEM images of (a) SiO_2_ nanospheres and (b) CeO_2_ nanospheres, (c) their diameter distribution and (d) schematic diagram of the synthesis of the new polishing slurry.

Fig. 1 shows the TEM analysis of the abrasive particles and a schematic diagram of the synthesis of the novel environmentally friendly chemical mechanical polishing slurry. Firstly, the Stöber method^23,24^ was used to prepare silica. As shown in Fig. 1(a), the SiO_2_ nanospheres have good dispersibility without agglomeration and other impurities. After extensive particle size statistical analysis, it was concluded that the diameter of the silica is around 26 nm (Fig. 1(c)). In order to improve the polishing rate of CMP, a small amount of commercial ceria was added to the polishing solution. Fig. 1(b) shows a low-magnification TEM image of the CeO_2_ nanospheres and their diameter is about 15 nm. SiO_2_ nanospheres, CeO_2_ nanospheres, hydrogen peroxide (H_2_O_2_) solution with a concentration of 30 wt% and polyaspartic acid (PASP) were mixed to formulate the new polishing slurry. As depicted in Fig. 1(d), the CeO_2_ and SiO_2_ nanospheres were mixed in a mass ratio of 1 : 100 and dissolved in a solution with a mass fraction of 6%. Simultaneously, PASP was dissolved in a solution with a mass fraction of 0.45%. Meanwhile, the 30 wt% H_2_O_2_ was diluted to 6 wt%. The three solutions were uniformly mixed in a volume ratio of 1 : 1 : 1. Lastly, sodium bicarbonate was used to adjust the pH to about 7.5. As is well-known, the uniformity and shape of the abrasive particles in the polishing slurry play a crucial role in their polishing effect.^25^ The structural characteristics of the abrasive particles in the CMP slurry reported in this study are better than those of previously reported materials.^26^ The material removal rate (MRR, μm h^−1^) can be calculated by the following equation:^16^1
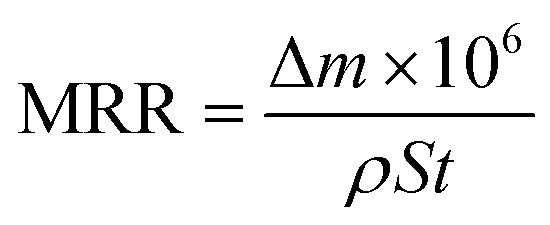
where Δ*m* is the mass loss of copper wafers after polishing (g), *ρ* is the density of the copper wafers (8.9 g cm^−3^), *S* is the total contact area between the polishing pad and copper substrates (mm^2^) and *t* is the polishing time (h). The Δ*m* is 4.27 mg. The total contact area *S* is 100 mm^2^. The time of CMP is 20 min. During CMP, the MRR calculated from eqn (1) was 14.4 μm h^−1^.

**Fig. 2:**
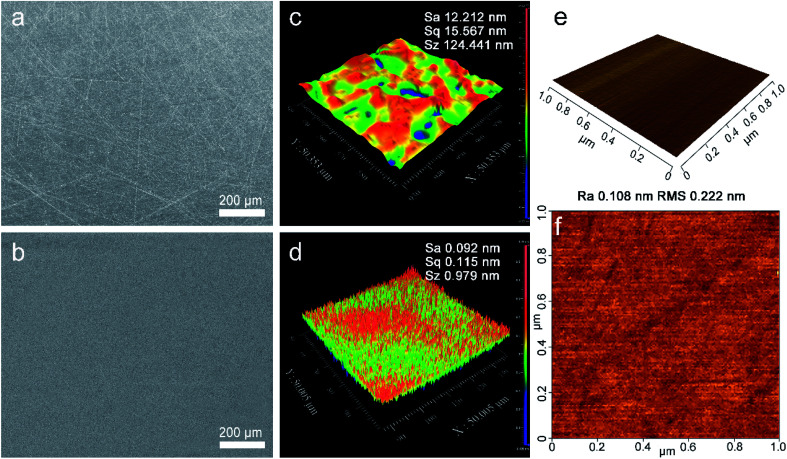
SEM images on the surface of the (a) lapped and (b) polished OFC wafers; surface roughness measured by the Zygo profilometer of the (c) lapped and (d) polished OFC wafers. (e) 3D and (f) 2D AFM images of the polished surface.

The developed polishing slurry was applied to the polishing of OFC wafers. At first, the wafers were lapped with abrasive paper as a pre-polishing treatment. Fig. 2 illustrates the surface topographies and surface roughness of the OFC wafers after lapping and CMP. After lapping, there were deep scratches on the surface; moreover, the surface was embedded with abrasive particles (Fig. 2(a)). However, after polishing, the scratches were removed and the rough surface became extraordinarily smooth (Fig. 2(b)). Fig. 2(c) depicts the surface roughness of the OFC wafers lapped by sandpaper with a grit size of 4000. The area roughness parameters Sa (arithmetical mean height), Sq (root mean square height) and Sz (maximum height) were 14.565 nm, 20.543 nm and 407.438 nm, respectively, over a measurement area of 200 × 200 μm^2^. At the same time, the polishing surface roughness was characterized with a Zygo profilometer. The area roughness parameters Sa, Sq and Sz were 0.092 nm, 0.115 nm and 0.979 nm, respectively, over a measurement area of 50 × 50 μm^2^ (Fig. 2(d)), which are much lower than those reported in previous studies.^27^ Compared with the surface roughness of lapping, the roughness of polishing is improved by 2 orders of magnitude. As far as we know, the surface roughness Sa values of the polishing surface in this study are the lowest values reported to date. Simultaneously, to make the experimental results more convincing, AFM was employed to measure the surface topographies of the polishing wafers. Fig. 2(e) shows a three-dimensional (3D) view of the CMP surface and Fig. 2(f) shows a 2D or planar view of the surface roughness for the CMP surface. The average roughness (Ra) and root mean square (RMS) values of the CMP surface are found to be 0.108 nm and 0.222 nm, respectively. According to reports in the literature, the diameter of the copper atom is 0.264 nm (ref. 28) and the interplanar spacing of the Cu (111) plane is 0.21 nm.^29^ Therefore, the obtained OFC surface achieved atomic-level flatness.

**Fig. 3:**
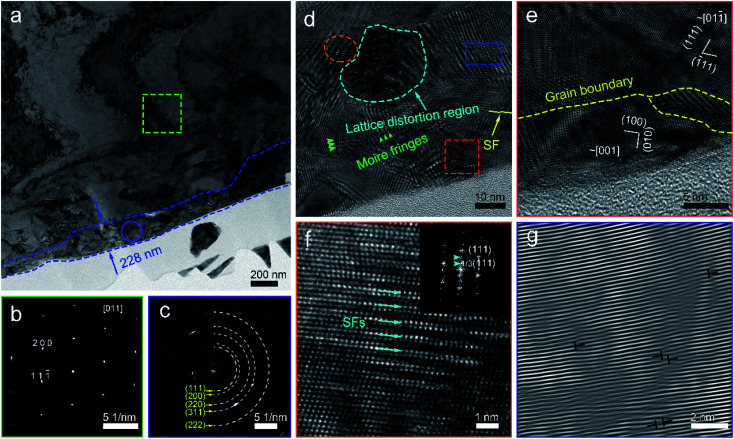
(a) A low-magnification cross-sectional TEM image of the lapped wafer surface; SAED pattern taken from the area of (b) the green square and (c) the purple circle in (a). (d) Enlarged high-resolution transmission electron microscopy (HRTEM) image of the abrasive damaged area and its magnified areas shown by (e) a red square and (f) an orange ellipse. (g) IFFT image of the area marked by a blue rectangle in (a). The inset in (f) shows the FFT image of the superlattice.

To understand the mechanical removal mechanism of the abrasive particles during lapping and CMP, the lapping and polishing surfaces and subsurfaces were analyzed. Fig. 3 shows the TEM characterization and corresponding selected area electron diffraction (SAED) patterns of the lapped wafer surface. The thickness of the damaged layer on the OFC wafer is 228 nm (Fig. 3(a)). Silver conductive paint (SCP) was used to protect the abrasive surface from ion beam processing during the preparation of the TEM samples. Prior to lapping, the pristine OFC wafer is highly single-crystalline (Fig. 3(b)). After lapping, it was found that the SAED pattern turns polycrystalline (Fig. 3(c)). These results indicate that the lapping process can bring about grain breakage. To better understand the damage from the lapping process, the damaged layer was enlarged and analyzed. At the topmost region of the damaged layer, due to high abrasive stress, the single-crystal was broken and obvious grain boundaries appeared, as marked by the yellow dotted line in Fig. 3(e). However, in the inner region of the damaged layer, with the reduction of abrasive stress, moiré fringes and lattice distortion regions, marked by green arrows and cyan dotted lines, respectively, appeared sequentially (Fig. 3(d)). The moiré patterns were formed by the overlapping and intersection of two crystalline grains,^30^ and the lattice distortion is one of crystal-structure defects.^31^ A superlattice structure adjacent to the lattice distortion region was formed under stress (Fig. 3(f)). Two columns of weaker spots marked by cyan arrows (inset of Fig. 3(f)) indicated there is a superperiod that is three times the interplanar spacing of the (111) plane. The inverse fast Fourier transform (IFFT) image, whose area is marked by a blue rectangle in Fig. 3(d), shows the presence of edge dislocations at the right side of the lattice distortion region (Fig. 3(g)). In summary, the damaged layer consists of a lattice distortion region, moiré fringes, grain boundary, superlattice and edge dislocations. In the superficial region of the damaged layer, the damage layer is mainly composed of grains; however, in the inner region of the damaged layer, the damage layer is mostly composed of crystal defects.

**Fig. 4:**
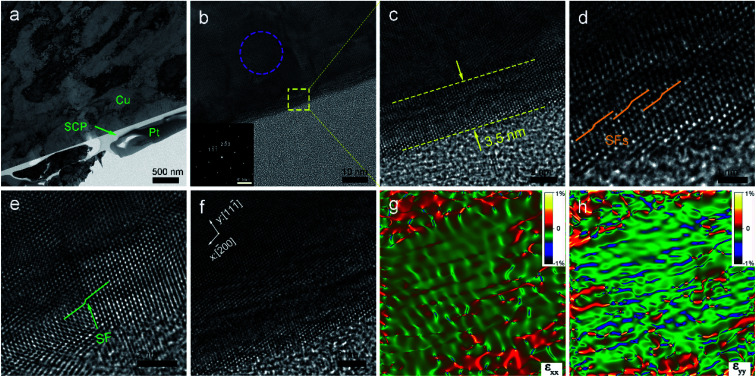
(a) A low-magnification cross-sectional TEM image of the CMP wafer surface; (b) enlarged HRTEM image of the CMP-damaged area and its magnified areas shown by (c) a yellow square; crystal defects ((d), (e) and (e)) in different damaged regions; a HRTEM image of the lattice distortion and its corresponding strain field *ε*_xx_ (g) and *ε*_yy_ (h) after CMP obtained by GPA. The inset in (b) shows the SAED pattern of the undamaged area.

After CMP, a TEM cross-section was prepared using FIB technology. As shown in Fig. 4(a), the SCP clung to the polished copper wafer surface and it could contribute to identifying the damaged surface. In the interior undamaged region of the Cu wafer, copper still remained as a single-crystal (inset of Fig. 4(b)). With further magnification of the damaged area, the thickness of the damaged layer on the polished OFC wafer was found to be 3.5 nm (Fig. 4(c)). Compared with the lapping-damaged layer, the CMP-damaged layer was reduced by 2 orders of magnitude. Meanwhile, the damaged areas at different locations were characterized. There was a spot of stacking faults with single-layer atoms inside the crystal lattice (Fig. 4(d)). Fig. 4(e) shows the core structure of a stacking fault (SF), and the length of the SF was about 2.4 nm. CMP technology can remove the material residual stress on the material surface,^32^ but it also generates processing stress. Microstrain analysis was thus performed on the HRTEM image by geometric phase analysis (GPA).^33,34^ The variation of strain was analyzed along the [11−1] and the [−200] directions (Fig. 4(f)). In the strain maps, the blue zones were under strong compressive strain; the red ones and the green ones represented strong tensile strain and no strain, respectively. The strain variation was found along the [11−1] direction. Nevertheless, there is no significant stress distribution along the [−200] direction (*ε*_xx_ in Fig. 4(g)). It should be noted that there is a large amount of weak compressive stress between the (11−1) planes (*ε*_yy_ in Fig. 4(h)). The above results reveal that CMP technology can generate processing stress and that compressive stress exists in a direction parallel to the wafer surface.

**Fig. 5:**
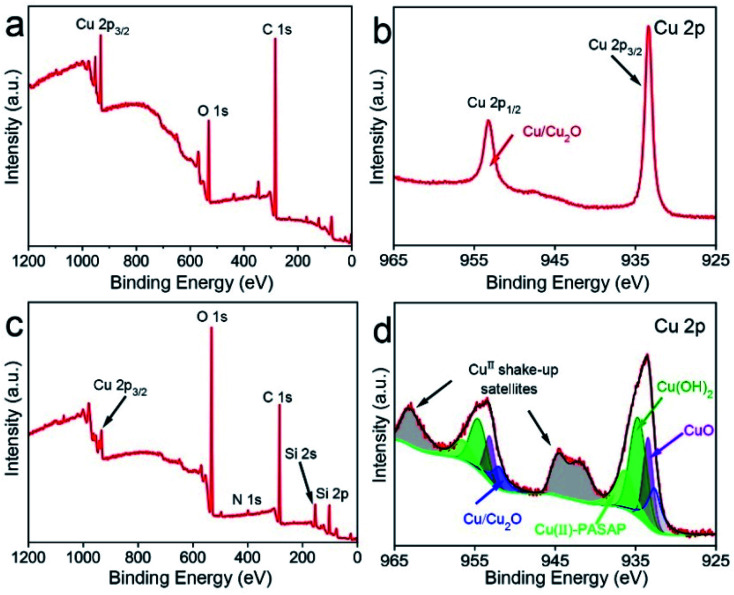
XPS spectra of the copper surfaces obtained prior to CMP: (a) survey and (b) Cu 2p spectra; and after CMP: (c) survey and (d) Cu 2p spectra.

**Fig. 6:**
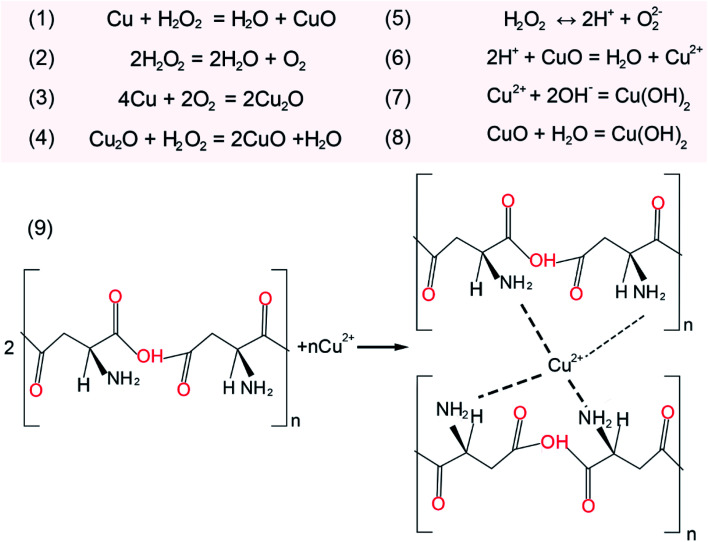
The chemical reaction equations during CMP.

To investigate the chemical mechanism in the course of CMP, X-ray photoelectron spectroscopy (XPS) was performed to study the surface composition and chemical state of the OFC wafers. The peak positions were calibrated with C 1s at 284.8 eV. As shown in Fig. 5(a), the XPS survey spectra indicate that there were Cu and O elements on the copper surfaces prior to CMP. Fig. 5(b) shows the Cu 2p high-resolution spectrum with double peaks of Cu 2p, which correspond to Cu 2p_3/2_ located at a binding energy of 932.4 eV and Cu 2p_1/2_ located at a binding energy of 952.1 eV. These peaks were attributed to Cu/Cu_2_O.^35^ These results confirm that there is a thin native oxide layer on the surface of copper. After CMP, the intensity of the oxygen (O) element increased obviously and peaks of Si and N appeared, as shown in Fig. 5(c). This indicated that the Cu element on the surface was severely oxidized. Meanwhile, the peak of the Si element was located at around 103.5 eV, which was assigned to the binding energy of Si–O–Si bonds.^36^ This indicated there is SiO_2_ on the surface of the CMP copper, which resulted from the abrasive particles in the polishing slurry. As for the peak of the N element, it might be derived from PASP. Additionally, in the Cu 2p binding energy region (Fig. 5(d)), the Cu 2p_3/2_ region fitting resulted in four distinguishable peaks located at 932.4 eV, 933.5 eV, 934.4 eV and 936.2 eV. These positions agree well with the known binding energy values for Cu/Cu_2_O, CuO, Cu(OH)_2_ and Cu(NH_2_)_4_^2+^ bonds, respectively.^16,37,38^ The peaks corresponding to Cu 2p_1/2_ were located at the binding energies of 952.1 eV, 953.1 eV, 954.7 eV and 956.6 eV, with satellite peaks observed at 944.5 eV and 963.2 eV. In the initial stage, this was due to chemical oxidation-dominant polishing. H_2_O_2_ and O_2_ in the polishing slurry resulted in oxidized layers (CuO and Cu_2_O) on the copper surface. From the above XPS results, the oxidation reaction has been proposed in Fig. 6 (eqn (1)–(4)).^39^ The hardness of CuO (∼2 GPa (ref. 40)) is similar to that of the OFC (1.8 GPa (ref. 41)) and the hardness of SiO_2_ is 9.03 GPa.^42^ Hence, the abrasive particles can easily remove the oxidation layer. Since the copper wafers were in a weakly alkaline environment during CMP, the wafers were also chemically corroded. The corrosion reaction equations can be concluded as shown in Fig. 6 (eqn (5)–(8)).^43,44^ In addition, polyaspartic acid had a chelating effect on metal ions. Cu^2+^ ions were chelated in the copper surfaces. Eqn (9)^45,46^ presents the chelating reaction between Cu^2+^ and PASP molecules (Fig. 6).

**Fig. 7:**
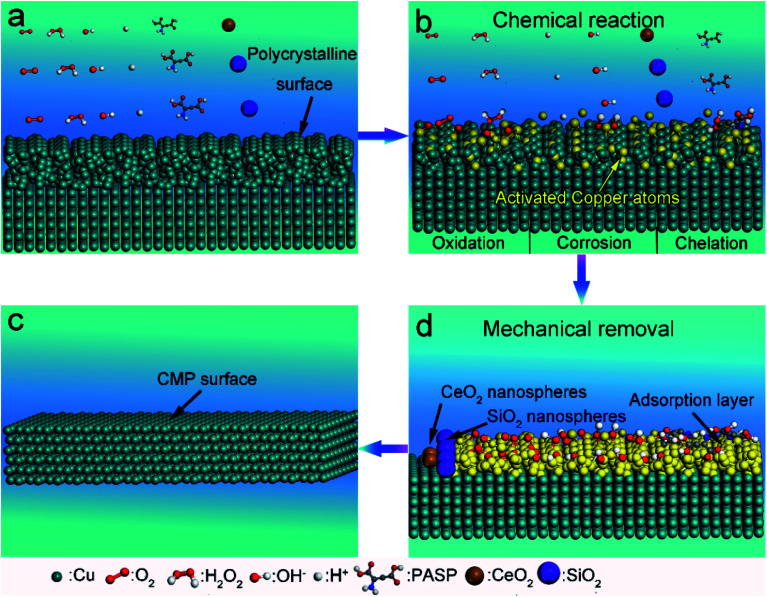
Schematic of the CMP mechanism: (a) the original surface before CMP; (b) the chemical reaction, (c) mechanical removal and (d) flat surface after CMP.

As mentioned above, during the CMP process, a complex chemical reaction takes place on the copper surface. Summarizing the above information, the CMP mechanism was proposed. Initially, before CMP, the surface was rough and composed of a polycrystal layer (Fig. 7(a)). First, the bonds of the copper atoms were broken and the copper atoms were in an active state. Oxygen atoms bonded with the activated copper atoms and copper oxides (*i.e.* CuO, and Cu_2_O) were generated. At the same time, copper atoms were also corroded by H^+^ ions. Next, Cu^2+^ ions were chelated by PASP molecules and bonded with OH^−^ ions (Fig. 7(b)). Ultimately, the adsorption layer (hydroxide and chelate) generated by the chemical reactions was removed by the nanospheres (Fig. 7(d)). In the end, atomic-level flatness was acquired (Fig. 7(c)).

## Conclusions

In summary, homogeneously distributed SiO_2_ nanospheres have been prepared and a novel CMP slurry consisting of SiO_2_ and CeO_2_ nanospheres, PASP, hydrogen peroxide, sodium bicarbonate and deionized water has been developed for OFC. The CMP slurry is employed to polish OFC wafers. After CMP, atomic-level flatness on the OFC surface is acquired. The lapping and CMP mechanisms are systematically proposed according to TEM and XPS characterization. Firstly, during lapping, under the effect of grinding stress, there is a severely damaged layer consisting of a lattice distortion region, moiré fringes, grain boundary, superlattice and edge dislocations. During CMP, the OFC is first oxidized by H_2_O_2_ and O_2_. At the same time, hydroxide and hydrogen ions are released by H_2_O_2_ and sodium bicarbonate, respectively, and the oxide layer is corroded. Next, the Cu^2+^ ions are bonded with PASP. Finally, the soft layer is removed by silica nanospheres. This study provides practical guidance for the subsequent improvement of the CMP of OFC.

## Author contributions

Dongdong Liu and Zhenyu Zhang developed the CMP experiment and wrote the paper. Zhenyu Zhang and Wei Liu conceived of the project. Dongdong Liu and Zhibin Yu analyzed the data. All authors discussed the results and commented on the manuscript.

## Conflicts of interest

The authors declare that they have no known competing financial interests or personal relationships that could have appeared to influence the work reported in this paper.

## Supplementary Material
